# Lanreotide Depot: An Antineoplastic Treatment of Carcinoid or Neuroendocrine Tumors

**DOI:** 10.1007/s12029-016-9866-9

**Published:** 2016-09-13

**Authors:** Edward M. Wolin, Amandine Manon, Christophe Chassaing, Andy Lewis, Laurent Bertocchi, Joel Richard, Alexandria T. Phan

**Affiliations:** 1 Medical Oncology, Montifiore Einstein Center for Cancer Care, 1695 Eastchester Rd., Second Floor, Bronx, NY 10461 USA; 2 Clinical Pharmacokinetics and Drug Metabolism, Ipsen Innovation, 5 Avenue du Canada, 91140 Les Ulis, France; 3 Peptide Development, Beaufour Ipsen Industrie, Rue Ethe Virton, 28109 Dreux, France; 4 Houston Methodist Hospital, 6410 Fannin St, Houston, TX 77030 USA

**Keywords:** Lanreotide, Depot preparations, Neuroendocrine tumors, Drug formulation

## Abstract

**Purpose:**

Peptide drugs for antineoplastic therapies usually have low oral bioavailability and short in vivo half-lives, requiring less preferred delivery methods. Lanreotide depot is a sustained-release somatostatin analog (SSA) formulation produced via an innovative peptide self-assembly method. Lanreotide is approved in the USA and Europe to improve progression-free survival (PFS) in patients with unresectable gastroenteropancreatic neuroendocrine tumors (GEP-NETs) and also approved in Europe for symptom control in carcinoid syndrome associated with GEP-NETs. This review discusses how the distinct molecule and formulation of lanreotide depot provide advantages to patients and health care providers, as well as the most recent clinical evidence demonstrating the safety and efficacy of lanreotide depot in inhibiting tumor growth and controlling hormonal symptoms in GEP-NETs.

**Methodology and Results:**

The lanreotide depot formulation confers a remarkable pharmacokinetic profile with no excipients, comprised only of lanreotide acetate and water. Of note, lanreotide depot constitutes an example for peptide self-assembly based formulations, providing insights that could help future development of sustained-release formulations of other antineoplastic peptides. Most patients with GEP-NETs will present with inoperable or incurable disease; thus, medical management for symptoms and tumor control plays a crucial role. Recent long-term clinical studies have demonstrated that lanreotide depot is well tolerated, prolongs PFS in GEP-NET patients, and significantly reduces symptoms related to carcinoid syndrome.

**Conclusions:**

The unique depot formulation and delivery method of lanreotide confer advantages in the treatment of metastatic GEP-NETs, contributing to improvements in NET-related symptoms and PFS without reducing quality of life in this patient population.

## Introduction

Peptides are attractive therapeutic molecules due to their significant activity and low toxicity profile. It is not surprising that the number of peptide-based drugs being evaluated in clinical trials has been steadily increasing over the years. Currently, many peptide drug candidates are in clinical or preclinical development phases, and many are being evaluated in the oncology setting [1]. However, peptide drugs usually have low and variable oral bioavailability and short in vivo half-lives. Therefore, they require delivery methods such as infusion, frequent injections, implantable devices, or intramuscular injection of large drug volumes, which are not always preferred by health care providers and patients [2, 3].

Fortunately, an intrinsic biophysical property of some peptides may help overcome the problems associated with peptide drug delivery. Under specific conditions of pH, concentration, and salts, many peptides and proteins will self-assemble into filamentous structures such as hollow nanotubes or densely packed fibers that have all the properties of an ideal sustained-release formulation [2, 4]. Firstly, they only contain the densely packed peptide (commonly as an acetate salt) and water, which enable very small injection volumes even for high doses. Secondly, they form highly organized and stable structures. The active drug, i.e., the peptide, is very stable in these structures and is released in a controlled manner over an extended period of time. This stability also enables sustainable supply and storage of prefilled, ready-to-inject syringes. Furthermore, release of the active drug from the depot can provide a rapid response [2].

Lanreotide is an analog of human somatostatin, and lanreotide depot (Somatuline Depot; Ipsen Pharma SAS, Paris, France) is the first marketed sustained-release formulation produced via peptide self-assembly. Lanreotide depot has been used by patients with acromegaly for more than 13 years [5] and has recently been approved in the USA for the treatment of patients with unresectable, well- or moderately differentiated, locally advanced, or metastatic gastroenteropancreatic neuroendocrine tumors (GEP-NETs) to improve progression-free survival (PFS) [6]. Lanreotide depot has also been approved in Europe since 2003 for symptom control in carcinoid syndrome associated with GEP-NETs [5].

NETs are a group of malignancies that arise from the secretory cells of the neuroendocrine system. NETs can develop in virtually any organ but are predominantly found in the gastrointestinal system, particularly the pancreas and gastrointestinal tract [7]. GEP-NETs are rare; however, the age-adjusted incidence of GEP-NETs has been steadily increasing, with a 3.6-fold increase observed between 1973 and 2007 [7–9]. Improved disease awareness and improved diagnostic imaging technologies [10] may play a role in the apparent increase in incidence [7]. Based on the most updated data from the Surveillance, Epidemiology and End Results program of the National Cancer Institute, the incidence of GEP-NETs was 3.65/100,000 individuals per year between 2003 and 2007 [9].

While indolent in nature, advanced well-differentiated NETs remain incurable with overall poor prognosis, and only a few treatment options can effectively inhibit their growth [11–13]. Goals of therapy in managing patients with GEP-NETs are twofold: symptoms and tumor control. The majority of NETs are nonfunctioning, i.e., they do not secrete excess hormones that cause clinical symptoms. This lack of symptoms in nonfunctioning NETs may delay diagnosis until after the disease has advanced and metastasized, when patients will exhibit symptoms relating to their bulky/advanced disease burden. Conversely, functioning NETs are characterized by excessive hormone secretion resulting in distinct clinical syndromes, depending on the hormone secreted. A common example is the carcinoid syndrome, which is caused by systemic release of serotonin and tachykinins by gastrointestinal NETs and characterized by severe gastrointestinal symptoms including flushing and diarrhea [11, 13]. Functioning and nonfunctioning NETs are histologically indistinguishable as they are clinically defined/classified, and goals of treatment remain the same—controlling of tumor and controlling of symptoms (from hormone over-secretion or from bulky tumor burden).

In most patients with advanced or metastatic GEP-NETs, curative surgery may not be an option, and systemic management is an important part of clinical care. Somatostatin analogs (SSAs) play a prominent role in systemic management of GEP-NETs. GEP-NETs frequently overexpress receptors for the inhibitory hormone somatostatin, and SSAs have been shown to be effective in controlling symptoms of hormone hypersecretion in a number of functioning GEP-NETs [7, 13]. Furthermore, recent studies have shown that SSAs also have antiproliferative effects in GEP-NETs [12, 15].

This review discusses how the formulation of lanreotide depot provides potential advantages to both patients and health care providers, as well as the most recent clinical evidence demonstrating the safety and efficacy of lanreotide depot in the control of tumor growth and hormonal symptoms in GEP-NETs.

## Lanreotide Depot Formulation

The first long-acting formulation of lanreotide (Somatuline LA, Ipsen Pharma) comprised a polymeric microsphere system and was intended for dosing every 7, 10, or 14 days. Polymeric microsphere-based formulation systems have been shown to be effective and have also been used in other long-acting SSAs. However, microsphere-based formulations have inherent disadvantages, including the need for reconstitution before injection, a complex manufacturing process requiring the use of organic solvents that are often toxic, potential burst release of the drug, the generation of acidic metabolites during the polymer degradation process, and possible degradation of the peptide [3, 16–18]. Moreover, microsphere-based formulations often necessitate potentially painful intramuscular injections as well as large injection volumes that can limit the amount of drug administered at a time and hence the length of drug exposure. Thus, an innovative approach was required to develop a new formulation for lanreotide with an improved pharmacokinetic (PK) profile and free of the aforementioned disadvantages.

**Fig. 1:**
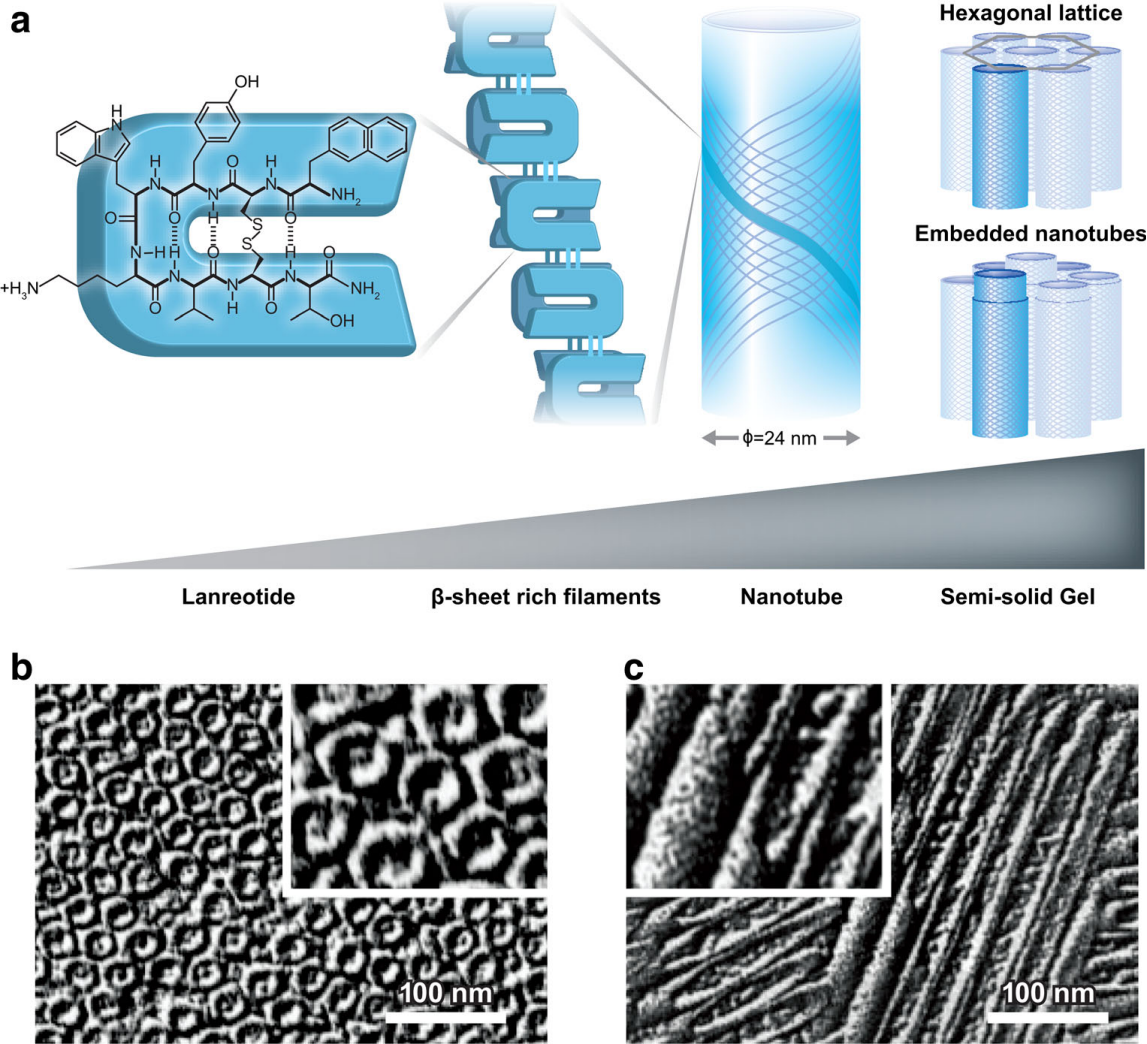
**a** Lanreotide is a cyclic octapeptide that can form noncovalently bonded dimers. The dimers constitute β-sheet-rich filaments that are stabilized by hydrogen bonding. Twenty-six of the filaments make up the hollow nanotubes, which have a highly uniform diameter of 24 nm. Depending on the lanreotide concentration, nanotubes in the semi-solid gel can be organized in a hexagonal lattice or form densely packed tube-within-a-tube structures (embedded nanotubes). Freeze-fracture electron micrographs of lanreotide in water (14 %, *w*/*w*), with the plane of fracture **b** perpendicular or **c** parallel to the direction of nanotubes. *Insets* are 2× enlarged. *Scale bar* = 100 nm [25–27]. **b**, **c** Reprinted from [25]

Studies on lanreotide formulation led to a remarkable discovery that the peptide self-assembles into a gel under certain conditions. After extensive work led by Cherif-Cheikh and Valery et al. [19–28], the self-assembly process and liquid crystal structure of lanreotide depot formulation are now well characterized. The process starts with noncovalent peptide dimerization, formation, and growth of an open ribbon and ends with ribbon closure in the shape of a nanotube (Fig. 1a) [23]. In the nanotube, dimeric building blocks form β-sheet-rich filaments that constitute the hollow nanotubes with a highly uniform diameter and wall thickness. The structure of the semi-solid gel results from a very dense packing of the nanotubes. At lanreotide concentrations of up to 10 to 15 % (*w*/*w*), the semi-solid gel is primarily composed of an organized hexagonal lattice of nanotubes (Fig. 1b, c), whereas the nanotubes start to form a densely packed tube-within-a-tube structure (“embedded nanotubes”) at higher lanreotide concentrations (Fig. 1a) [25, 26]. The nanotubes are stabilized by noncovalent forces including hydrogen bonding between dimeric subunits, hydrophobic interactions, and π-π stacking of the aromatic side chains within the filaments (Fig.1a) [27]. These interactions can form and be maintained in water, without the need for any other substance or excipient. An understanding of the biophysical forces involved in nanotube assembly will help future development of self-assembly-based peptide formulations. Most importantly, in vitro experiments have shown that the nanotube assembly is completely reversible. At low lanreotide concentration, the nanotubes slowly disassemble, releasing the active drug [19, 23]. The slow and controlled drug release that starts immediately upon transfer into a dilute solution renders the nanotube formulation an ideal candidate for fast-acting and sustained delivery of lanreotide.

**Fig. 2:**
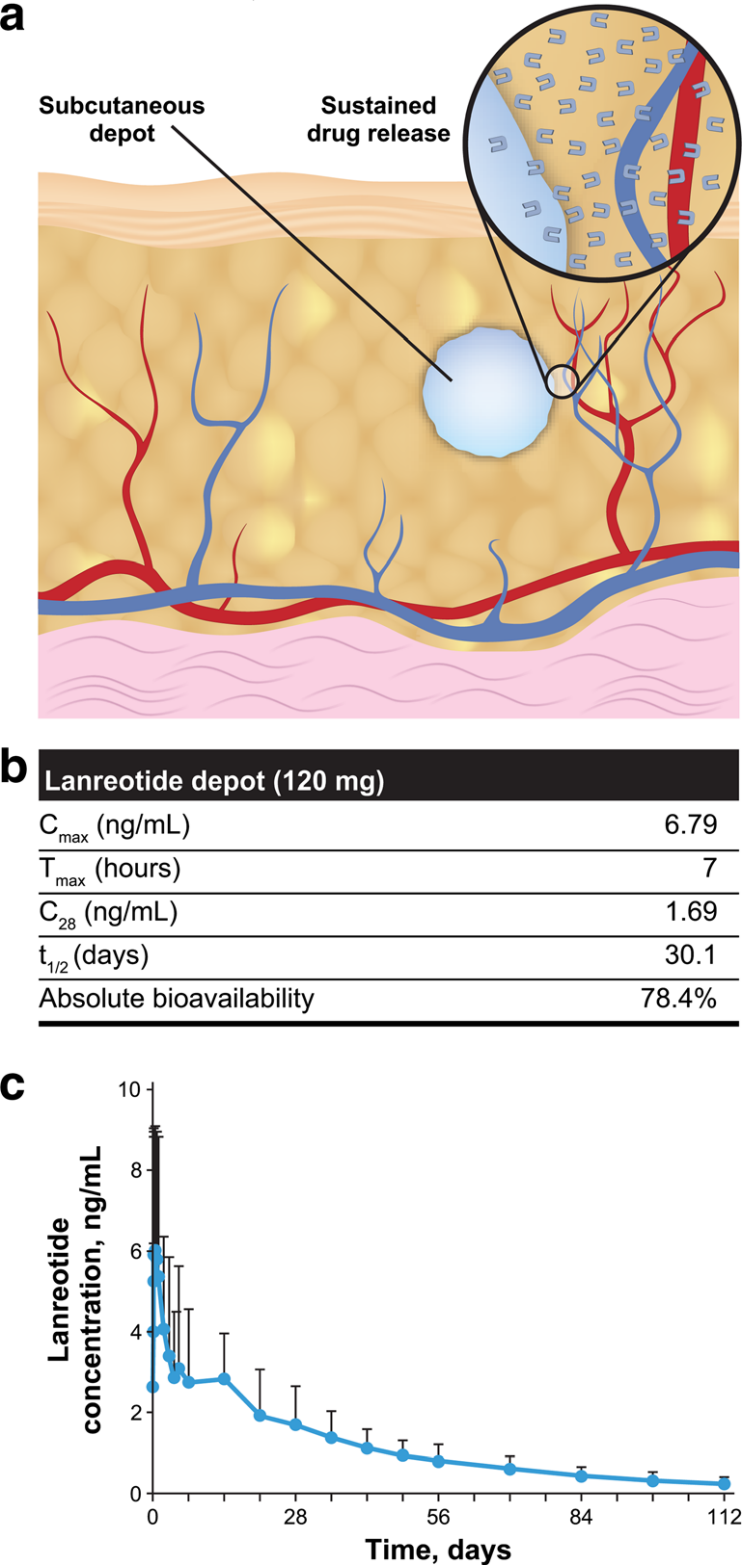
**a** Schematic view of subcutaneous depot and sustained drug release. *Inset* shows diffusion of active drug to the adjacent capillaries. **b** Pharmacokinetic properties and **c** concentration-time curve of lanreotide depot after a single 120-mg injection in healthy volunteers [5, 6]. *C*_*28*_ mean serum concentration 28 days after injection, *C*_*max*_ peak serum concentration, *T*_*max*_ time to reach *C*_max_, *t*_*1/2*_ apparent terminal elimination half-life

In vivo, lanreotide is believed to form a depot at the injection site, based on the interaction of the formulation with physiological fluids. In vivo release of lanreotide is governed by the intrinsic properties of self-assembly of the peptide itself and is most likely mediated by passive diffusion of lanreotide from the depot into the surrounding tissues, followed by the absorption to the bloodstream (Fig. 2a) [6, 29]. Indeed, PK studies confirmed the favorable PK profile expected based on the previous data. When a small volume of concentrated lanreotide self-assembly formulation (120 mg, 0.5 mL) was injected subcutaneously in healthy volunteers, it formed a rapid-acting depot (Fig. 2a), with significant lanreotide concentrations reached within the first day as demonstrated by the short time to reach peak serum concentration (*T*_max_, 7 h). The subcutaneous depot provided a controlled, sustained release of the drug over extended periods of time with a low peak serum concentration (*C*_max_, 6.79 ng/mL) and long terminal half-life (30.1 days). The mean serum concentration 4 weeks after injection was 1.69 ng/mL (Fig. 2b, c) [5, 6]. PK properties were similar in patients with acromegaly, and a linear PK profile between the 60- and 120-mg doses was observed [30, 31]. In patients with acromegaly, the mean (SD) steady-state trough serum lanreotide concentrations in patients receiving lanreotide depot every 4 weeks were 1.8 (0.3), 2.5 (0.9), and 3.8 (1.0) ng/mL at 60-mg, 90-mg, and 120-mg doses, respectively [6, 30]. In patients with GEP-NETs who received a 120-mg dose of lanreotide depot every 4 weeks, steady-state concentrations were reached after four to five injections, and the mean trough serum lanreotide concentrations at steady state were 5.3 to 8.6 ng/mL [6]. The PK properties of lanreotide depot formulation render it suitable for a 4-week dosing interval [6].

The depot formulation also confers significant advantages in terms of administration, such as enabling sustained supply and storage of ready-to-use prefilled dosing devices, which eliminates the need for a multistep reconstitution process. It also allows small-volume subcutaneous administration of the drug [6]. Moreover, a new injection device for lanreotide, which has recently been approved in the USA, provides additional safety advantages including a rigid needle cap, automatic needle guard to prevent needlestick injuries, and a fully transparent syringe to help confirm successful delivery [32].

The potential advantages of lanreotide depot formulation and the new injection device were assessed in a quantitative opinion study conducted with 77 nurses in Europe and the USA; all of whom had previous experience with SSAs [32]. To evaluate the administration of two long-acting SSAs in clinical practice (lanreotide depot [subcutaneous] and octreotide LAR [intramuscular; Sandostatin LAR; Novartis, Basel, Switzerland]), the participants were asked to score both drugs in terms of 16 administration-related attributes, and lanreotide depot received a significantly higher overall preference score (114 vs 70, *P* < 0.01) (Table 1). The only attribute for which the drugs received a similar preference score was the sturdiness of the plunger (Table 1). Confidence that a full dose had been delivered was the most important attribute according to the nurses. Indeed, improved reliability and consistent delivery of accurate drug doses may have a positive effect on observed drug efficacy, particularly in patients who receive care in a community setting. Moreover, easy and quick administration, as well as the low risk of clogging, could help conserve time and resources [33].

**Table 1:** Nurse’s preference with self-assembly formulation [32]

Attribute	Mean relative importance score (scale of 1–10)	Mean preference score (scale of 1–10)	
		Octreotide LAR	Lanreotide depot
Confidence that a full dose has been delivered	9.5	6.8	9.5
High product efficacy	9.4	7.5	8.6
Good safety	9.4	5.8	8.9
Low risk of clogging	8.8	3.7	9.4
Easy/convenient preparation and injection	8.8	3.8	9.4
Easy to teach	8.3	4.4	9.1
Low risk of needlestick injuries	8.2	4.9	8.8
Prefilled device	8.1	3.1	9.5
Calm environment for patient	7.9	6.4	7.9
Fast administration (preparation & injection)	7.9	3.7	9.3
Sturdy plunger	7.9	7.7	7.5
Transparent device	7.8	7.8	9.2
Comfortable device to hold	7.7	6.3	7.6
Short/thin needle	7.6	3.8	7.1
Depth of injection (IM vs deep SC)	7.2	5.6	8.1
Self-injection possible	6.8	3.2	8.4
Overall preference score		*70*	*114*

Nurses rated a predefined list of attributes on a scale of 1 to 10 (1 = not important/not preferred, 10 = most important/most preferred). Lanreotide depot (new device) received higher preference scores in all attributes except for the sturdiness of the plunger. Overall preference score, which included scores in all 16 attributes weighted by relative importance, was significantly higher with lanreotide depot (*P* < 0.01)

*IM* intramuscular, *SC* subcutaneous

Subcutaneous delivery of lanreotide depot, which was received favorably in the aforementioned nurse opinion study (Table 1) [32], may offer important advantages in ease and accuracy of administration. For instance, intramuscular injections can be associated with significant risk of unintended subcutaneous placement [34], particularly in obese patients because of the thick layer of adipose limiting access to muscle tissue or in cachectic patients who have lost significant muscle mass [35]. The high rate of improper intramuscular injections may reduce the effectiveness of the medicine in a community setting and increases the potential risk of tissue damage [34, 35]. Moreover, intramuscular SSA injections are more commonly associated with injection-site pain than are subcutaneous SSA injections [36].

Further studies are needed to confirm the findings of the nurse opinion study in real-world practice and assess the potential of lanreotide depot formulation to meet expectations of both health care providers and patients.

## Efficacy and Safety of Lanreotide Depot in NETs

For decades, SSAs have played a significant role in the management of hormonal symptoms in metastatic GEP-NETs [5, 37]. Lanreotide is approved for the treatment of carcinoid syndrome in more than 50 countries but not yet in the USA. In light of recent clinical evidence including the landmark study CLARINET, lanreotide depot is now also approved by the US Food and Drug Administration (FDA) for the treatment of patients with unresectable, well- or moderately differentiated, locally advanced GEP-NETs to improve PFS [6]. In this section, three recent clinical trials evaluating the efficacy and safety of lanreotide depot in the treatment of GEP-NETs will be discussed.

### CLARINET: Antiproliferative Effect in Advanced GEP-NETs

CLARINET was a 96-week, randomized, double-blind, placebo-controlled, parallel-group, international, phase 3 study in 204 patients with advanced, well- or moderately differentiated, nonfunctioning, metastatic, and/or locally advanced, unresectable, grade 1 or 2 (Ki-67 < 10 %) GEP-NETs.

The primary end point was PFS, defined as the time to disease progression or death. Secondary end points included overall survival, quality of life, and safety [12].

**Fig. 3:**
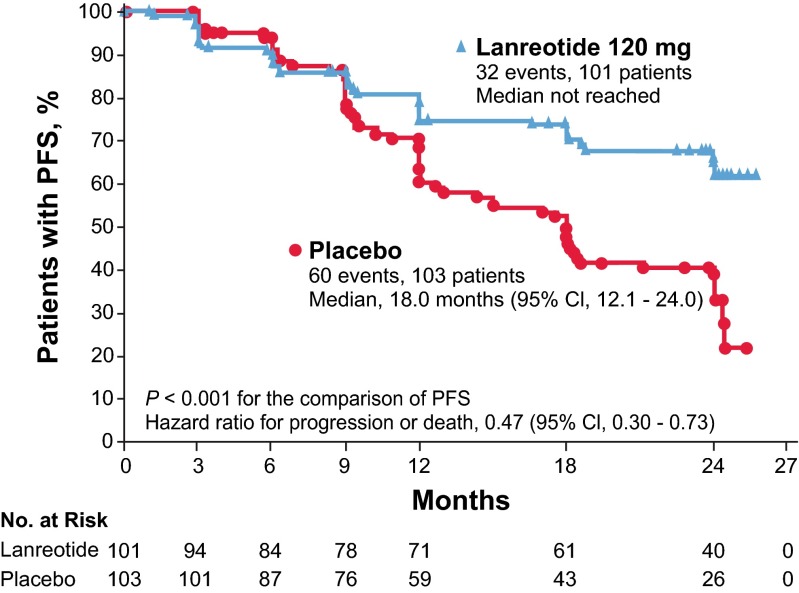
Estimates of PFS among patients who received lanreotide depot (120 mg) or placebo in the 96-week, placebo-controlled CLARINET core study. Kaplan-Meier curves were compared using a stratified log-rank test. Stratification was based on the presence or absence of tumor progression at baseline and the receipt or nonreceipt of previous therapy. The hazard ratio was derived from a Cox proportional hazards model with terms for study treatment, the presence or absence of tumor progression at baseline, and the receipt or nonreceipt of previous therapy [12]. *CI* confidence interval, *PFS* progression-free survival. From [12] (reprinted with permission from Massachusetts Medical Society)

CLARINET demonstrated that lanreotide depot 120 mg administered subcutaneously once every 4 weeks significantly improved PFS in patients with advanced, well- or moderately differentiated, somatostatin receptor-positive GEP-NETs, regardless of hepatic tumor volume [12]. In patients who received lanreotide depot, median PFS was not reached and was 18.0 months in patients who received placebo (*P* < 0.001). The risk of disease progression or death within 96 weeks of first dose was significantly reduced with lanreotide (hazard ratio 0.47; 95 % confidence interval [CI], 0.30–0.73) (Fig. 3) [12]. At week 96, estimated rates of PFS were 65 % in the lanreotide depot group and 33 % in the placebo group. At both weeks 48 and 96, the percentage of patients alive without disease progression was higher in the lanreotide depot group (Table 2) [12]. The PFS benefits were observed in almost all predefined subgroups regardless of tumor origin (midgut, pancreas, other), tumor grade (grade 1 or 2), and hepatic tumor volume (>25 or ≤25 %) [12]. A significant effect of lanreotide depot on overall survival was not evident, possibly due to relatively long life expectancy in patients with slow-growing tumors [38]. In addition, crossovers from placebo to lanreotide depot and treatments received after progression may have complicated the analysis [12]. Among patients with higher-than-normal baseline levels of NET biomarker chromogranin A, a more than or equal to 50 % reduction in chromogranin A levels was achieved in 42 % of patients treated with lanreotide depot compared with only 5 % in the placebo group (Table 2) [12].

**Table 2:** Secondary efficacy end points in CLARINET core study (intent-to-treat population) [12]

End point	Lanreotide depot (*n* = 101)	Placebo (*n* = 103)	*P* value
Patients alive without disease progression^a^			
Week 48	66 %	49 %	<0.05
Week 96	52 %	25 %	<0.001
Median time to tumor progression (95 % CI), months^b^	Not reached	18.0 (12.1 to 24.0)	<0.001^c^
Patients with ≥50 % reduction in level of chromogranin A from baseline to last post-baseline level available^d^	42 %	5 %	<0.001

*CI* confidence interval

^a^The measures of progression-free survival shown here are different from the primary end point of CLARINET in that they are measurements at discrete time points and with patients counted as having treatment success or failure at each assessment

^b^There were 30 events in the lanreotide group and 58 events in the placebo group

^c^The between-group difference was analyzed with the use of the log-rank test

^d^Data are shown for the subgroup of patients with a baseline chromogranin A level higher than the upper limit of the normal range (98.1 μg/L) who also had evaluable data after baseline (64 patients in each group). Calculations were made with the use of a logistic-regression model with terms for study treatment and receipt or nonreceipt of previous therapy

In accordance with the accumulated data demonstrating safety of lanreotide depot in other indications including long-term experience in patients with functioning NETs [6, 39], lanreotide depot was generally well tolerated in CLARINET [12]. Despite the relatively long median exposure time (24.0 months), lanreotide depot treatment did not increase the proportion of patients who had adverse events (AEs) (88 % compared with 90 % with placebo), and most of the AEs experienced in both groups were mild or moderate. The most common study-related AEs were gastrointestinal related and included diarrhea (26 %) and abdominal pain (14 %) (Table 3) [12]. In addition, lanreotide depot treatment did not cause deterioration in the quality of life as assessed by the European Organization for Research and Treatment of Cancer Quality of Life Questionnaire-Core 30 (EORTC-QLQ-C30) global health status score [12].

**Table 3:** Summary of AEs in CLARINET core study (safety population) [12]

Event	Lanreotide depot (*n* = 101)	Placebo (*n* = 103)
	No. of patients, %	
Any AE	89 (88)	93 (90)
Any AE related to study treatment	50 (50)	29 (28)
Any AE according to intensity^a^		
Severe	26 (26)	32 (31)
Moderate	44 (44)	44 (43)
Mild	17 (17)	17 (17)
Study treatment-related AEs in ≥5 % of patients		
Diarrhea	26 (26)	9 (9)
Abdominal pain	14 (14)	2 (2)
Cholelithiasis	10 (10)	3 (3)
Flatulence	8 (8)	5 (5)
Injection-site pain	7 (7)	3 (3)
Nausea	7 (7)	2 (2)
Vomiting	7 (7)	0
Headache	5 (5)	2 (2)
Lethargy	5 (5)	1 (1)
Hyperglycemia	5 (5)	0
Decreased level of pancreatic enzymes	5 (5)	0

AEs were defined according to the Medical Dictionary for Regulatory Activities, version 16.0

*AE* adverse event

^a^For patients with multiple AEs, events with the maximum intensity are shown; data are missing for two patients in the lanreotide group

A total of 88 patients from the core study (41 from the lanreotide group, 47 from the placebo group) participated in the open-label extension of CLARINET. A median PFS of 32.8 months was reached for the lanreotide group during the extension. No new safety concerns were identified [40].

A study in 85 patients with low-grade (grade 1), midgut NETs demonstrated that octreotide LAR (30-mg intramuscular injection, every 4 weeks) prolongs time to tumor progression in the studied patient population (PROMID study) [15]. This study supports the body of literature regarding the activity of SSAs as antiproliferative agents in NETs. However, octreotide LAR is not FDA-approved as an antiproliferative agent to improve PFS in GEP-NETs [37].

Although CLARINET included only patients with nonfunctioning tumors, earlier open-label studies of lanreotide depot have suggested activity in all patients with GEP-NETs regardless of tumor-functioning status [41, 42]. The presence of a functional hormone secretion syndrome is not relevant in terms of antiproliferative treatments, and tumor grade as well as somatostatin receptor expression status are among the most important factors that determine response to SSAs in GEP-NETs [13, 14]. Accordingly, lanreotide depot is approved in the USA for the treatment of both functioning and nonfunctioning GEP-NETs [6].

In summary, CLARINET showed that lanreotide depot prolonged PFS in patients with advanced, grade 1 to 2, somatostatin receptor-positive GEP-NETs regardless of hepatic tumor volume.

### ELECT and SYMNET Studies: Symptom Control in Carcinoid Syndrome

**Fig. 4:**
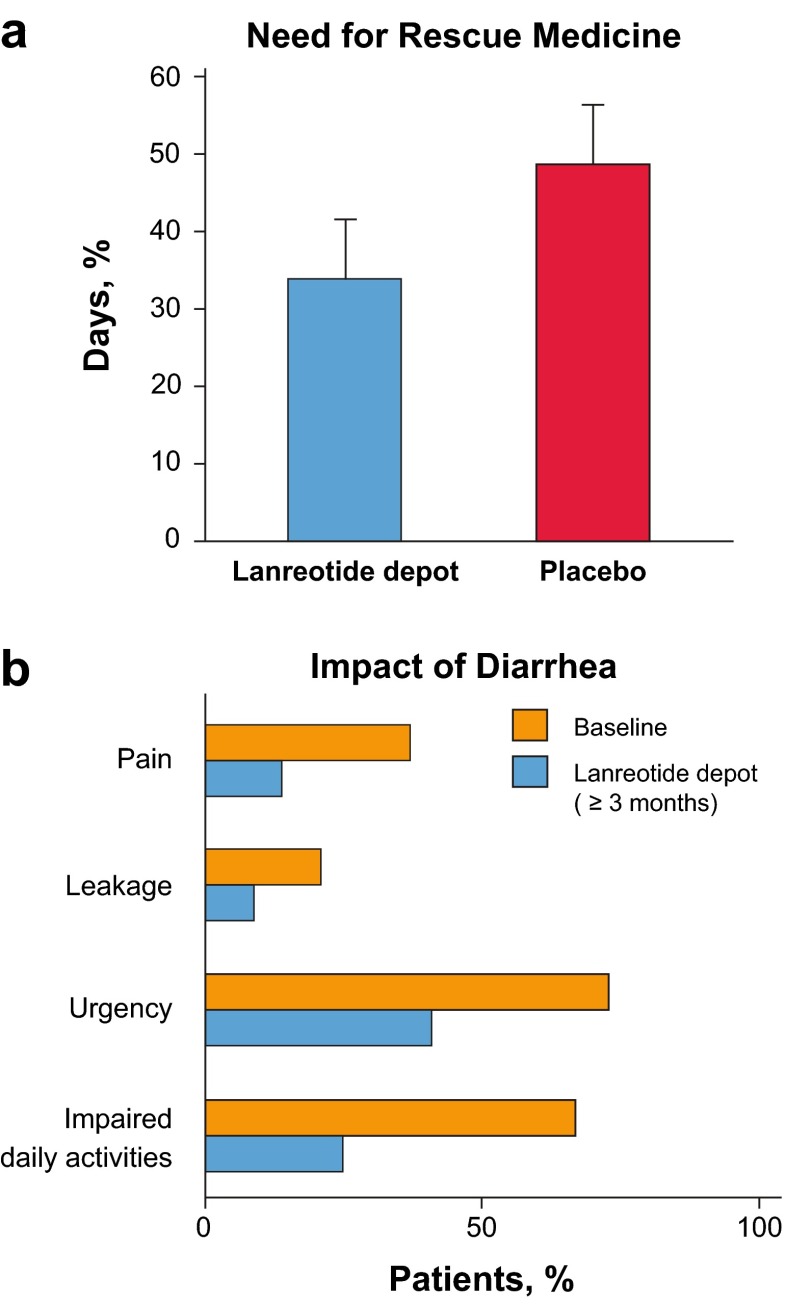
**a** In the ELECT study, mean percentage of days with rescue medication use was statistically lower with lanreotide depot (34 [95 % CI, 25–42]) vs placebo (49 [95 % CI, 40–57]) (*P* = 0.02). *Error bars* denote 95 % CI. **b** In the SYMNET study, the impact of diarrhea on patients was reduced with lanreotide depot (≥3.0 months) treatment. There were statistically significant reductions in the numbers of patients with diarrhea-related pain (37 vs 14 %), leakage (21 vs 9 %), and urgency (73 vs 41 %) (McNemar paired tests, *P* < 0.001). Fewer patients had impaired daily activities due to diarrhea during treatment (25 %) than before (67 %) [43, 44]. *CI* confidence interval

Two recent studies have evaluated the efficacy of lanreotide depot in control of hormonal symptoms associated with carcinoid syndrome: ELECT and SYMNET [43, 44]. The phase 3 ELECT study included a 16-week, double-blind, placebo-controlled phase and a 32-week open-label extension phase and was conducted in SSA-naive (44 %) and octreotide-responsive (56 %) patients (*n* = 115). Patients were randomized to receive subcutaneous lanreotide depot 120 mg every 4 weeks or placebo. Because assessment of symptom control in carcinoid syndrome has always been a difficult study outcome to evaluate, a novel primary end point was used: mean percentage of days requiring rescue medication (MPDR). Patients were allowed to use a rescue medication (short-acting octreotide; Sandostatin; Novartis, Basel, Switzerland) to control breakthrough symptoms throughout the study, and MPDR was significantly lower in lanreotide depot vs placebo arms, at 34 vs 49 % (*P* = 0.017, Fig. 4a). The primary end point was met, and this represented a clinically significant difference of approximately 5 days less use of a rescue medication on the lanreotide depot arm. Moreover, complete or partial success, defined as ≤3 days use of rescue medication per week, was achieved significantly more commonly with lanreotide depot compared with the placebo group (odds ratio 2.4; 95 % CI, 1.1–5.3) [43]. Treatment-emergent AEs were observed in 26 % of patients in the lanreotide depot group compared with 19 % with placebo. Most AEs were not serious, and only one patient from each group discontinued the treatment due to AEs. The most frequent AEs were gastrointestinal [43].

SYMNET was a large (*n* = 273), multinational, cross-sectional, observation study to evaluate patient satisfaction with symptom control during lanreotide depot treatment in patients with GEP-NETs who experienced carcinoid syndrome-related diarrhea [44]. At routine clinical visits, patients who had been receiving lanreotide depot for at least 3.0 months completed questionnaires. Satisfaction with diarrhea control was the primary end point. The majority of patients (79 %) reported improved diarrhea control with lanreotide depot, and most patients (76 %) were completely or rather satisfied with this effect. The satisfaction regarding the control of flushing was similarly high (73 %). Compared with baseline, a clinically significant decrease in median daily stool frequency (from 4 to 2) was observed. The overall impact of diarrhea on patients was also significantly lower than baseline, as evidenced by reduced pain, leakage, urgency, and impact on daily activities (Fig. 4b). The ELECT and SYMNET studies confirmed that lanreotide may help patients sustain good control of carcinoid syndrome symptoms.

## Conclusions

Interest in peptide-based antineoplastic drugs has been increasing; however, administration of these drugs has historically posed significant challenges in terms of sustaining a therapeutic dose over a long period of time. Peptide self-assembly-based formulations provide an innovative solution to these challenges.

Lanreotide depot is a chemically distinct SSA and the first marketed drug with a self-assembly-based sustained-release activity. Extensive research on the lanreotide peptide self-assembly process provides insights that could help future development of self-assembly-based formulations for therapeutic peptides or the bottom-up design of new peptide families that self-assemble into complex architectures with desired properties.

SSAs have been used for the treatment of symptoms associated with carcinoid syndrome, and now, based on evidence from a phase 3 study, lanreotide depot is the first, and currently the only, SSA that is approved in the USA for the treatment of metastatic GEP-NETs to improve PFS. Moreover, lanreotide depot’s formulation and method of delivery provide the potential to improve quality of life and tumor management in patients with GEP-NETs.
